# Construction of an Yucatec Maya soil classification and comparison with the WRB framework

**DOI:** 10.1186/1746-4269-6-7

**Published:** 2010-02-13

**Authors:** Francisco Bautista, J Alfred Zinck

**Affiliations:** 1 Centro de Investigaciones en Geografía Ambiental, Universidad Nacional Autónoma de México, Antigua Carretera a Pátzcuaro No. 8701, Col. Ex-Hacienda de San José de La Huerta, C.P. 58190 Morelia, Michoacán, México; 2 International Institute for Geo-Information Science and Earth Observation, PO Box 6, 7500 AA Enschede, the Netherlands

## Abstract

**Background:**

Mayas living in southeast Mexico have used soils for millennia and provide thus a good example for understanding soil-culture relationships and for exploring the ways indigenous people name and classify the soils of their territory. This paper shows an attempt to organize the Maya soil knowledge into a soil classification scheme and compares the latter with the World Reference Base for Soil Resources (WRB).

**Methods:**

Several participative soil surveys were carried out in the period 2000-2009 with the help of bilingual Maya-Spanish-speaking farmers. A multilingual soil database was built with 315 soil profile descriptions.

**Results:**

On the basis of the diagnostic soil properties and the soil nomenclature used by Maya farmers, a soil classification scheme with a hierarchic, dichotomous and open structure was constructed, organized in groups and qualifiers in a fashion similar to that of the WRB system. Maya soil properties were used at the same categorical levels as similar diagnostic properties are used in the WRB system.

**Conclusions:**

The Maya soil classification (MSC) is a natural system based on key properties, such as relief position, rock types, size and quantity of stones, color of topsoil and subsoil, depth, water dynamics, and plant-supporting processes. The MSC addresses the soil properties of surficial and subsurficial horizons, and uses plant communities as qualifier in some cases. The MSC is more accurate than the WRB for classifying Leptosols.

## Background

Ethnoecology is concerned with studying the relationships between humans and nature, and investigates how indigenous people perceive, know and use the landscapes and their natural resources. This approach puts emphasis on the cultural value of the belief-knowledge-practice (kosmos-corpus-praxis or K-C-P) complex [1]. Ethnopedology, as part of ethnoecology, seeks to explore the connections, synergies and feedbacks between symbols, concepts and perceptions of soils and soilscapes in local societies [2-5].

Yucatec Maya have used soils over four millennia, providing a good example for understanding soil-culture relationships. The soils occurring in the Maya territory have been well documented [6-14]. For instance, Pérez [7] describes soil profiles in the southern portion of the Yucatán state, using the FAO soil classification adapted to the Mexican context [15]. This study is the first one recognizing the Maya soil reference groups (MRGs) of Ek' lu'um, Yax kom and Ak'al che', and their local uses. Using chemical and physical topsoil properties, Pool and Hernández [8] highlight important short-distance differences between the MRGs of Ho lu'um and K'an kab lu'um in the eastern part of the Yucatán state. Duch [16,17] reports a variety of Maya soil-related names from the southern Yucatán state. Working in the same region, Dunning [10] classifies the soils according to the USDA Soil Taxonomy [18], the INEGI soil classification system [15,19], and the Yucatec Maya soil nomenclature [17], but fails to analyze the differences among these soil classification schemes. Estrada [20] made a detailed description and sampling of 21 soil profiles in the Hocabá municipality, using the WRB classification [21] and the Maya nomenclature. This field information was subsequently used by Estrada et al. [22], together with local soil knowledge, to construct an indigenous soil classification and prepare a map using MRGs. Bautista et al. [12,13] studied micro-catenas in a karstic plain, highlighting the importance of using micro-relief features and soil color as diagnostic properties. They relate these features with chemical constituents, such as organic matter and phosphorus, and mineral contents of calcite, hematite, goethite, and boehmite. Bautista et al. [23] also highlighted the importance of soil-relief patterns in large areas within karstic plains for establishing a geopedologic map of the whole Yucatán state. In general, soil variability is controlled by relief and landforms from local and plot scales [12-14,24] to regional scales [25]. Using geostatistical analysis, Bautista et al. [14] showed the close correlation and complementarity of the numerical, Maya and WRB [21] classifications of 54 soil profiles from the Mérida municipality. The Maya soil, geoform and water knowledge at the Yucatán peninsula level was analyzed in an integrated way by Bautista et al. [24], implementing the K-C-P model as suggested by Barrera and Zinck [26] and Barrera and Toledo [1] to understand the Yucatec Maya ethnopedology.

The kosmos domain, which refers to the beliefs and symbolism associated with the indigenous culture, has been little studied in Yucatán [1,27]. Some studies report on the Maya experience (i.e., the praxis domain) in managing their soils [10,24,28,29]. Several studies have addressed the Maya soil corpus per se but only in small areas [12-14,17,22-24,29-33], and very few have attempted to compare the Maya soil nomenclature with the World Reference Base for Soil Resources [13,14].

The possibility of using indigenous soil knowledge for designing local soil classifications and amending international soil classifications is often questioned. Duch [17], for instance, considers that Maya soil names should be used only within the framework of the Maya soil nomenclature, while Krasilnikov and Tabor [4] sustain that folk systems are only locally valid and have relatively limited application compared to scientific systems. It is, however, remarkable that soil classifications were originally constructed from the farmers' knowledge. Dokuchaiev, for instance, documented and organized the soil knowledge of the Ukrainian peasants into a classification scheme [34]. Nowadays, the Maya soil nomenclature is used by more than 1.5 million people in the Yucatán peninsula.

The objective of this work was to organize the Maya soil nomenclature and knowledge and to construct a Yucatec Maya soil classification by comparison with the framework of the World Reference Base for Soil Resources.

## Methods

The relief in the Yucatán State, southeast Mexico, has developed from Miocene-Pliocene and Holocene limestones and includes, as main regional units, a coastal plain, a karstic plain, inland basins with hills (extended karst), and hillands crossed by valleys (tectono-karst) [35]. Our study was carried out mainly in the lowlands of the coastal and karstic plains.

The coastal plain is a strip of land very slightly inclined towards the sea that extends along the western and northern coast at less than 10 m above sea level. The climate is semiarid [36] and the vegetation cover is shrub, savannah and mangrove [37].

The karstic plain lies 10-60 m above sea level and its topography varies from horizontal to undulating. Two main geoforms, namely mounds and depressions, systematically recur throughout the landscape [12]. Mounds are lapiaz fields with large bedrock outcrops, intensively carved by minor solution channels, which dominate the depressions by a few meters elevation (2-10 m). Depressions are sinkholes (dolines) formed by solutional enlargement of joints and subsequent settling of the surface and/or by subsidence resulting from roof collapse of small caverns. In general, shallow black soils occur on mounds and deep red soils in depressions. Climate is subhumid warm with summer rains [36]. The most common vegetation cover is dry forest [37].

The inland territory of the peninsula has also been formed by karstification and includes basins with isolated hills and larger hilly relief units crossed by valleys. Hills reach elevations of about 220 m above sea level, while basins and valleys are flat, closed depressions at 120-150 m above sea level [25].

Forty-five open interviews were conducted between 2000 and 2009. In 2009, field trips with bilingual Maya-Spanish-speaking peasants took place. Some of these peasants were agricultural technicians from the Agroecology School "U Yits Ka'an" of Mani, Yucatán, who are knowledgeable with the main soils of the Yucatán state [13,25,29].

Structured interviews were not done because peasants do not feel comfortable when formal questionnaires are used. As a consequence, we missed the opportunity to perform statistical data analysis but responses gained in quality.

**Figure 1:**
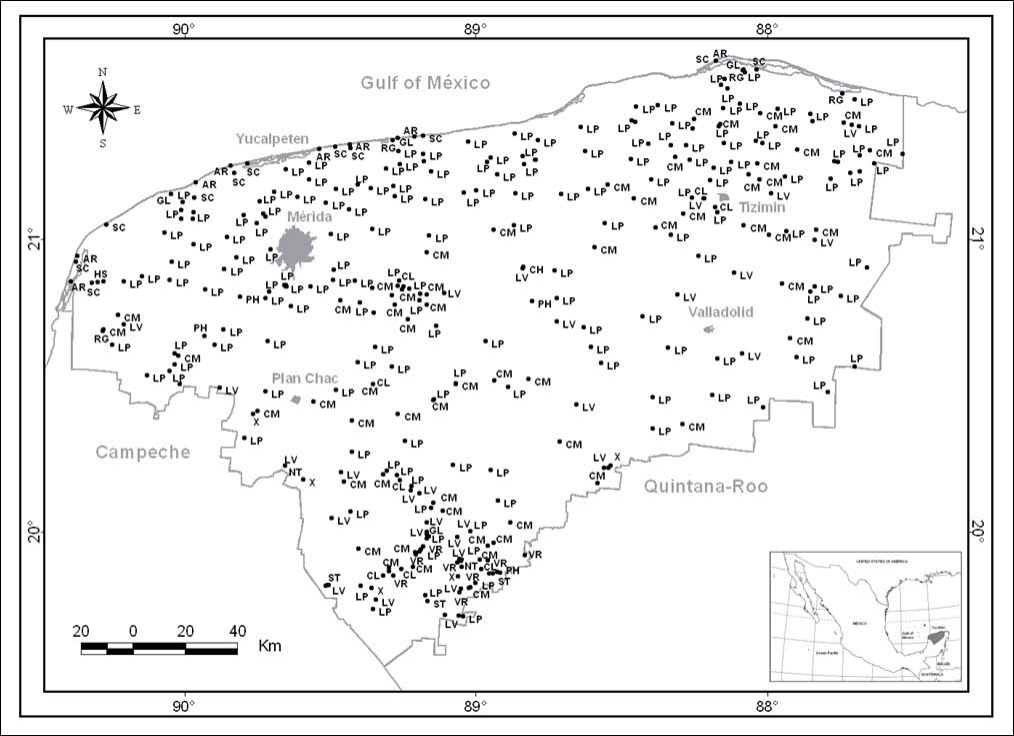
**Study area and location of soil profiles in the state of Yucatán**. LP = Leptosol, CM = Cambisol, LV = Luvisol, AR = Arenosol, GL = Gleysol, ST = Stagnosol, VR = Vertisol, NT = Nitisol and SC = Solonchack.

**Figure 2:**
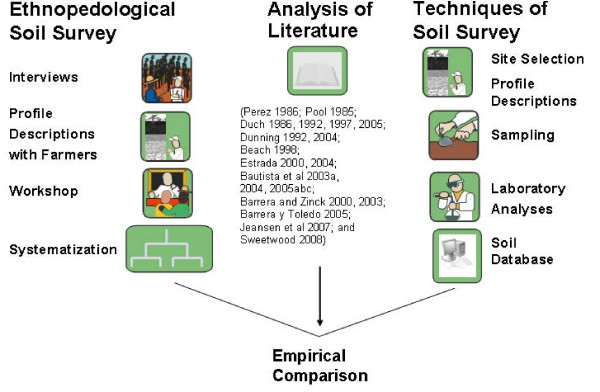
**Methodological approach**.

Soils were described and sampled at representative sites for laboratory analysis, and classified using the WRB [21]. A multilingual soil database was built with 315 soil profile descriptions, using the database structure developed by De la Rosa et al. [38] (Figure 1). By means of interviews, participative field transects and workshops, local farmers were asked to name and show the soil types, describe their properties, and explain the characteristics used to recognize them in the territory of their community (Figure 2).

The WRB framework was used to develop the MSC mainly because of its relatively simple structure that allowed accommodating the levels of soil perception shown by Maya farmers. It is also the international soil classification system most commonly used by Mexican soil scientists together with the national INEGI system. The WRB states comprising only two tiers of categorical information, but the practical operation of the framework implies four consecutive classification steps [21]. The system starts providing a set of ten classes based on soil properties, forming factors and processes, which serve as entries to the classification key. The following level, the most important of the system, includes 32 reference soil groups (RSGs) that are clustered into the ten entry classes aforementioned. Subsequently, soil classification is refined using a two-tier system of prefix (primary) qualifiers and suffix (secondary) qualifiers. Thus practically, a four-step procedure is used to classify a given soil in the WRB. We have implemented a similar categorical approach to construct the Maya soil classification scheme. The criteria used to define the entries to the classification key and the Maya soil reference groups (MRGs) are similar to those used in the WRB framework, namely in our case: (1) organic carbon content; (2) presence of features in the soil profiles that reflect strong anthropic influence; (3) physical restrictions to root growth; (4) water influence and drainage limitations; and (5) weak profile development (sandy soils). Additional criteria were extracted from the Maya soil nomenclature and implemented to subdivide the MRGs at lower levels. For instance, Maya people make a distinction between rock outcrops and stones as coarse fragments that hinder root development. Similarly, in Maya knowledge, the color contrast between A and B horizons is relevant to separate MRGs, probably as a reflection of differences in soil fertility or drainage. This distinction has important implications for planting strategies.

## Results

### Diagnostic soil properties

Maya peasants identify soil reference groups based on relief position, soil color, stoniness, rockiness, gravel content, depth, texture, structure and drainage, which are all soil properties of universal use in indigenous soil classifications [3]. Plant community and area size are also used as differentiating criteria in some particular sites. The MSC gives more weight to topsoil than subsoil properties. Many of these properties are also diagnostic attributes in scientific soil classifications, such as the WRB system and the USDA Soil Taxonomy [39].

The position of the soils on the terrain is a primary diagnostic feature [40]. Maya soil groups and soil units vary according to soil position on the landscape [13,23]. A major distinction takes place between soils on mounds (Ho-lu'um) and soils in depressions (Kankabal), the two main geoforms in the Yucatán karstic landscape. Also the word ka'anal lu'um designates soils on high sites [17]. While terrain position is used by Maya peasants for management purposes, it is considered mainly as a pedogenic factor in the WRB classification.

Color is usually taken as an accessory, co-variant soil property, as it reflects chemical and mineralogical properties that are not directly observable in field conditions, such as organic matter, iron and manganese contents, among others [41,42]. In the Yucatec Maya perception, color is a highly differentiating attribute used to distinguish soils at the higher levels of the soil classification. From the soils in the northern part of Yucatán, Bautista et al. [12,13] report a clear difference between the black soils on mounds and the red soils in depressions, the first ones being rich in organic matter, calcium and phosphorus, the second ones with high contents of Si, Al and Fe oxides, together with the presence of hematite and boehmite. Maya farmers use also color to distinguish key soil horizons. The concept of K'an kab, for example, means "yellow underneath" that refers to a yellow Bt horizon underlying a usually red epipedon in Luvisols.

Stoniness is a relevant property influencing soil productivity and soil management [43]. In karstic areas, the amount of coarse fragments in the soil reflects the intensity and stage of rock dissolution. High temperature and abundant rainfall accelerate the weathering of calcareous rocks, generating deep clayey soils, with neutral reaction and well developed structure [44,45]. Stoniness is an important differentiating property in the Yucatec Maya soil perception and classification. Special words are used to refer to stoniness (mulu'uch) and stone mounds (mu'ul). Particular MRGs (e.g., Ch'och'ol) allow distinguishing stony soils from others, which are strongly correlated with the Hyperskeletic Leptosols in the WRB classification [14]. The consideration given to stoniness in the MSC could help improve the WRB classification with the introduction of qualifiers to recognize the presence of calcareous coarse fragments in the Leptosols, such as Ch'ich'ic for gravelly soils and Ch'och'olic for stony soils.

Rockiness can take different forms that are reflected in two MRGs: (1) Chaltún soils on smooth laminar bedrocks with surface dissolution channels, and (2) Tzek'el soils on large, rugged promontories with cracks (karst mounds). In both cases, soils are poorly developed and very shallow, except along joints and fractures where limestone dissolution proceeds. Chaltún lu'um soils are extensive in the north of Yucatán under semiarid climate, with a thorny shrub cover and a variety of herbaceous plants that grow only during the short rainy season. To place these soils in the WRB system, Tzek'elic and Chaltunic are proposed as qualifiers of the Leptosols.

Depth is used as an indicator of effective soil volume. The MSC is more precise than the WRB classification, establishing a clear difference between Hay lu'um and Chaltún soils within the Lithic Leptosols. In Mayan language, different words are used to indicate soil depth, such as Hach taan lu'um for very deep soils; Taan lu'um and Taan taan lu'um for deep soils; Ma'taan lu'um for shallow soils; and Hach ma'taan taan lu'um for very shallow soils [17]. On the basis of depth criteria, the K'an kab lu'um soil class can be divided into three subgroups, resulting in a shallow (25-50 cm) K'an kab lu'um, a moderately deep (50-100 cm) K'an kab lu'um, and a deep (>100 cm) K'an kab lu'um. Recent modifications of the WRB [21] have led to eliminating depth limits as a diagnostic criterion, arguing that the latter are artificial and not genetic soil subdivisions. This is questionable in the case of the tropical karst in the Yucatán peninsula, where there are shallow soils that show degrees of development similar to those of deep soils [12,23,45]. We strongly support maintaining or re-introducing depth qualifiers, i.e., lithic in Leptosols, and epileptic and endoleptic in Kastanozems, as practical classes for farming purposes but also for morphological characterization.

Soil heterogeneity is relevant to farming. In the northern part of the Yucatán peninsula, soil distribution patterns are very complex, with frequent spatial variations at short distance. For example, Bautista et al. [14] identified six MRGs, corresponding to four types of Leptosol and one type of Kastanozem, on a surface area no larger than 1350 m^2^. This might be the reason why farmers integrate soil, land and soilscape in one comprehensive concept. By contrast, the southern part of the Yucatán state is more homogeneous. In the Pucc region, for instance, K'an kab lu'um, Chac lu'um, Ek' lu'um and Yaax kom, that are among the best soils of the peninsula, occupy in general large areas. Only Ak'al che' soils occur as small patches in swampy lowlands [28].

Yucatec Maya farmers use also the type and density of individual plants and plant communities as soil indicators. For instance, Ak'al che' are associated with hydrophytes, Chaltún lu'um with seasonal herbs, K'an kab lu'um and Chac lu'um with plants adapted to hydrophobic soil materials, and Tzek'el lu'um and Box lu'um with tree communities.

All this soil knowledge is integrated by farmers when it comes to crop selection and farming practices. Each soil class or soil unit is used according to its suitability for selected varieties of maize and other crops [46,47]. Engineering properties of soils were also taken into account when building pyramids [48].

### Soil nomenclature

The phonetic writing of the oral terms used by Maya peasants can lead to confusions. For example, the composite expression of Yaax kom lu'um means literally "the soil around a poorly drained area", while Yaax hom lu'um (with hom instead of kom) would mean "green soil". The apostrophes following consonants in Yucatec Maya words are used by linguists to indicate glottal stops. Thus, Ch'och'ol is preferable to Chochol, which in plain pronunciation has no meaning in Mayan language (Table 1).

**Table 1:** Yucatec Maya soil names

Maya	Spanish	English	References
*Chaltún*	Tierra donde hay lajas, con poca tierra encima	Soil with laminar bedrock	Bautista *et al*. (2003ab; 2005abc)
*Box lu*'*um*	Box: negro Lu'um: tierra	Black soil	Bautista *et al*. (2003ab; 2005abc)
*Pus lu*'*um*	Tierra seca, suave	Dry, soft soil	Barrera (1995); Dunning and Beach (2004)
*Ch*'*ich*'*lu*'*um*	Tierra con grava	Soil with gravel	Bautista *et al*. (2003ab; 2005abc), Duch (2005)
*Tzek*'*el lu*'*um*	Tierra con rocosidad tipo promontorio	Soil with large rock promontories	Dunning and Beach (2004)
*Ch*'*och*'*ol lu*'*um*	Suelo con piedras	Soil with stones	Duch (2005)
*K*'*an kab lu*'*um*	K'an: amarillo Kab: abajo	Yellow subsoil	Barrera (1995), Dunning and Beach (2004)
*Chak lu*'*um*	Chak: colorado Lu'um: tierra	Red soil	Barrera (1995)
*Ek*'*lu*'*um*	Tierra obscura, de las sabanas	Dark soil	Pérez (1984), Barrera (1995), Duch (2005)
*Yaax kom*	Yaax: antes Kom: valle, parte baja del terreno Tierras bajas	Land around low-lying terrain, around a swamp	Flores *et al*. (1994), Barrera (1995), Dunning and Beach (2004)

To distinguish among MRGs, Maya farmers give high weight to topsoil properties, in the same fashion as other indigenous people do in different agro-ecological zones [5]. However, in deep soils with contrasting morphology, they also take into consideration subsoil properties that influence soil management and/or crop adaptability. This is the case of the K'an kab lu'um soils that have red topsoil and yellow subsoil.

Soils enriched in organic matter from decomposition of human and animal wastes in earlier settlements, together with other rests of human activities such as ceramic shards and kitchen middens, are clearly distinguished from other kinds of soil and named Kakabb lu'um (Anthrosols). Similar soils have been described by Dunning and Beach [31], and Duch [17].

Incipient soils, poorly developed because of the prevailing environmental conditions, are frequent in the Yucatán peninsula. Shallow soils and soils with little fine earth material are segregated on the basis of vegetation cover density, water dynamics, and the degree of dissolution of the calcareous substratum. Tzek'el lu'um and Chaltún lu'um are rocky soils; Ch'och'ol lu'um and Box lu'um are stony soils; and Ch'ich' lu'um are gravelly soils.

The presence of calcareous coarse fragments is a dominant feature in the Yucatán soils and is recognized as such by the local farmers. Many national soil classifications (e.g., the French, German, Polish, and Russian) have specific groups to account for the occurrence of calcareous fragments in soils. The WRB classification, in contrast, does not fully recognize the essential role of calcareous rocks, stones and gravels in soils and excludes them from the Leptosols [39,49].

Tzek'el lu'um, Yaax kom and Ak'al che' are comprehensive concepts, referring simultaneously or alternatively to soils, soilscapes, lands, sites, ecosystems, or plant communities. For instance, Tzek'el lu'um designates the unproductive land and soilscape of Lithic Leptosols on mounds and in depressions. Yaax kom is a site name referring to the low-lying land that surrounds a swampy area. Ak'al che' is rather an ecosystemic concept, corresponding to a swamp with indicator trees such as *Dalbergia *sp., *Haematoxylon campechianum *L., *Bucida buceras*, and *Annona glabra *(Table 1). Akal means flooded area and ché means tree or vegetation. Thus, the combination of both particles in Ak'al che' refers to marshlands with soil seasonally flooded and covered with trees [9]. The term expresses the interaction between relief, hydrology and plant communities. The soils can be grey Gleysols or light brown Stagnosols. Ak'al che' is a good example to illustrate the indigenous land concept proposed by Ortiz et al. [50], where land is a specific terrestrial area that includes all attributes of the biosphere, directly observed in the topsoil or inferred from the presence of indicator plants or animals.

Maya peasants use soil names and other terms as modifiers to designate particular soils that share characteristics of several groups. Also Maya soil names can refer to soilscapes. For example, K'an kab Tzek'el is sometimes used for patches of shallow stony soils within a K'an kabal area. Pus ek' lu'um can be used for shallow transitional soils around a swath of deeper Ek' lu'um. Mulu'uch Tzek'el is sometimes used to reflect the essentially soil-less conditions found on some rocky mounds.

Maya use additional terms, not included in the classification scheme of Table 2, to refer to special soil or land conditions that significantly restrict their use potential. For example, Buy lu'um stands for poor soils, Sohol lu'um for dry and sterile soils, K'oha'an lu'um for degraded soils, and Ch'ech lu'um for compact soils [17,51].

**Table 2:** Soil descriptors of Maya reference groups and correspondence with WRB soil groups

Soil descriptors	MSC	WRB
Black soils with abundant organic matter, fresh litter and litter in decomposition, in wet areas generally covered by mangrove	*Pu*'*uc lu*'*um*	Histosols
Black soils with high content of organic matter derived from human and animal wastes (former homegardens), containing also potsherds, ash, and other domestic residues	*Kakkabb lu*'*um*	Hortic Anthrosols
Black soils, with very little fine earth, bedrock outcrops in the form of promontories, stones >25 cm diameter	*Tzek*'*el lu*'*um*	Lithic Leptosols
Black soils, with little fine earth, soft, shallow, >10% organic matter, well drained, high water retention, with or without calcium carbonate, laminar limestone	*Pus lu*'*um*	Lithic Leptosols, Rendzic Leptosols, Mollic Leptosols
Light gray soils, sandy clay loam, extremely shallow (3-17 cm), poorly drained, calcareous over laminar limestone	*Sak lu*'*um*	Gleyic Lithic Leptosols (Calcaric)
Predominant rock outcrops of laminar limestone, large amounts of coarse fragments, with very little fine earth of red, reddish-brown or black color	*Chaltún*	Nudilithic Leptosols
Very shallow soils (<10 cm), red, reddish-brown or black, 3-15% organic matter, <50% stones, few rock outcrops	*Hay lu*'*um*	Lithic or Nudilithic Leptosols
Black soils, with more fine earth than *Tzekel *soils, >90% stones, coarse fragments >5 cm diameter	*Ch*'*och*'*ol lu*'*um*	Hyperskeletic Leptosols
Black soils, shallow (<25 cm), >90% gravel, >10% organic matter, high water retention	*Ch*'*ich*'*lu*'*um*	Hyperskeletic Leptosols
Black soils, with little fine earth, shallow, 20-60% gravel and stones, >10% organic matter, well drained, with or without calcium carbonate	*Box lu*'*um*	Mollic Leptosols
Grey or red soils, deep (>100 cm), clayey, no stones, temporary cracks, hard when dry	*Yaax kom lu*'*um*	Haplic Vertisols
Red soils, deep (>100 cm), clayey, no stones, temporary cracks, hard when dry, fertile (>50% exchangeable bases)	*Yaax kom- K*'*an kab lu*'*um*	Haplic Vertisols (Chromic)
Grey soils, moderately deep (<100 cm), clayey, temporary cracks, no stones, no rocks, swampy during the rainy season, in agricultural lands and large areas	*Yaxx kom-Ak*'*al che*'	Gleyic Vertisols
Grey soils, temporarily flooded, moderately deep (<100 cm), clayey, temporary cracks, no stones, no rocks, swampy in summer, fall and winter, plant community with *Dalbergia *sp. and *Haematoxylum campechianum*	*Ak*'*al che*'*grey*	Gleysols
Light brown soils, temporarily flooded, moderately deep (<100 cm), clayey, temporary cracks, no stones, no rocks, swampy in summer, fall and winter, plant community with *Bucida burceras*	*Ak*'*al che*' light brown	Stagnosols

### Proposed classification scheme

**Figure 3:**
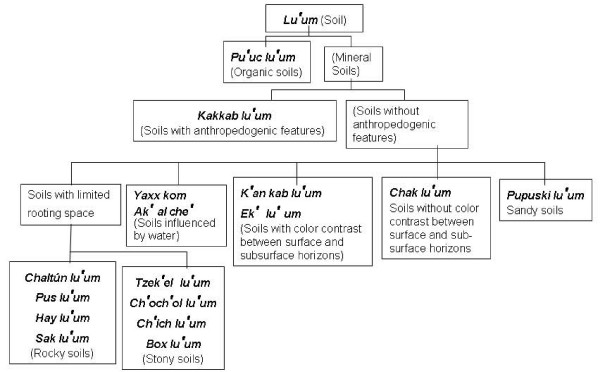
**Yucatec Maya soil classification scheme**.

On the basis of the diagnostic soil properties and soil nomenclature used by Yucatec Maya farmers, we have constructed a folk soil classification scheme with a hierarchic, dichotomous and open structure based on the WRB framework. Maya soil properties were used at the same categorical levels as similar diagnostic properties are used in the WRB system (Figure 3).

The first division is between organic and mineral soils to separate the Pu'uc lu'um soils (Histosols), which occur in areas of the karstic plain neighboring the coastal plain. The second division considers the presence of anthropedogenic features to separate Kakkab lu'um soils that are found in all regional relief units. Kakkab lu'um are homegarden soils (Hortic Anthrosols) that are enriched in organic matter derived from human and animal wastes but may also contain potsherds, ceramic shards, ash, and other domestic residues. Their location allows tracing former human settlements.

All other mineral soils that do not show conspicuous anthropedogenic features are grouped in five classes on the basis of rockiness/stoniness, water influence and drainage conditions, color contrast between topsoil and subsoil, and the occurrence of sandy texture.

(1) Soils with limited rooting space because of rockiness and/or stoniness at shallow depth. These soils are separated on the basis of the same criteria as those used in the WRB. Rock fragments can be boulders as in Tzek'el lu'um or laminar limestone slabs as in Sak lu'um, Pus lu'um, Chaltún and Hay lu'um. Tzek'el lu'um (Lithic Leptosols) occur mainly on mounds and hillslopes in all regional relief units, while Sak lu'um (Gleyic Lithic Leptosols) are common in the coastal plain (place of discharge of the groundwater). Pus lu'um are found in small areas, usually of less than one hectare, in all regional relief units. The Pus lu'um concept covers a variety of soils including Lithic Leptosols, Mollic Leptosols and Rendzic Leptosols, reflecting variability in soil depth, calcium carbonate and organic matter. Chaltún and Hay lu'um occur principally in the karstic plain, near the coastal plain, but occasionally also in other relief units. The stony soils called Ch'och'ol and Ch'ich' lu'um are distributed in small areas of less than one hectare. Box lu'um are commonly shallow, well drained, black soils with little fine earth, 20-60% stoniness, >10% organic matter, and with or without calcium carbonate.

(2) Soils influenced by water and poor drainage conditions. These soils also are separated on the basis of the same criteria as those used in the WRB. Yaax kom and Ak'al che' are frequent in the south of the Yucatán peninsula. Yaax kom cover large areas in inland plains, while Ak'al che' are found in depressions between hills.

The central concept of Ak'al che' corresponds to soils temporarily flooded. These can be Gleysols as in Campeche or Stagnosols as it occurs sometimes in the southern Yucatán state. The difference between gleyic and stagnic properties is reflected in the vegetation cover. In the WRB system, Stagnosols were first considered "false Gleysols" mainly because of the lack of information for full characterization, but they have been recently separated from Gleysols as an individual group. Similarly, in the Maya soil classification, primary and secondary qualifiers are added to the central concept of the soil group. Thus, Ak'al che' soils can be either grey Gleysols or light brown Stagnosols.

(3) Soils with color contrast between surface and subsurface horizons. This soil class was built using the Maya perception of color contrast in well-developed and deep soils such as Luvisols and Phaeozems. K'an kab lu'um are widespread in the south of the penisula and occupy also small areas in the north. Deep Phaeozoms called Ek' lu'um occur in karstic depressions in the south.

(4) Soils without color contrast between surface and subsurface horizons. The absence of strong color contrast in less-developed mineral soils lacking B horizons is used by Maya to build a separate soil class. Chack lu'um are widespread in the karstic plains of the south and occur also in small areas in the north.

(5) Sandy soils. Pupuski lu'um are white sandy soils located in the coastal plain, with or without gleyic and/or salic properties. They can be distinguished from other grey or white soils occurring in the area (e.g., Sak lu'um) because they lack a lithic qualifier. Pupuski lu'um include Arenosols as well as Gleysols and Solonchaks. Thus the central concept of Pupuski lu'um can be specified using primary qualifiers for depth, gleyic properties, and salinity.

## Discussion

The relatively simple structure of the WRB helped us accommodate the levels of soil perception shown by Maya farmers. The criteria used in the WRB to distinguish entries to the classification key and reference soil groups were useful to construct the upper levels of the MSC scheme. The lower MSC levels are mainly based on the formalization of features used by the Maya for more detailed soil distinction.

The Maya soil classification can be used for improving the WRB and other soil classification systems, in particular in karstic landscapes. For instance, the Maya soil classification can provide qualifiers for Leptosols to cope with soil and landscape features that strongly influence land management and use, such as soil depth (e.g., extremely shallow soils), types of bedrock (e.g., promontory bedrock, laminar bedrock), surface and subsurface stoniness with ranges of size and quantity, and soil color. Stoniness and gravel content are relevant properties to build hierarchy in the Maya soil classification (e.g., Ch'och'ol and Ch'ich' lu'um). Rockiness can take different forms that are reflected in two MRGs: Chaltún soils have smooth laminar bedrocks with surface dissolution channels, while in Tzek'el soils bedrocks are large, rugged promontories with cracks. The WRB classification does not include this feature as a diagnostic property.

The Maya soil classification and the WRB classification are complementary. The MSC shares categories and classes with the WRB framework. This is an advantage for the scheme being understood by technicians and local scientists and being incorporated in specialized curricula at regional universities. It is recommended that both systems be used at a maximum level of detail, as together they provide valuable information on soil properties, distribution, formation, and use potential in the study area. The MSC is addressed especially to extension agents and other experts involved in rural development as a means for communicating with Maya farmers in terms of soil management, farming practices and crop selection.

The soil properties used to build the MSC agree with similar soil properties used in indigenous soil classifications in other parts of the world [3,5,11]. As indigenous soil classification schemes are mental constructs, resulting from the way the soil scientist interprets farmers' soil perceptions, variations might appear among the schemes proposed by different authors to organize the Yucatec Maya soil knowledge [11].

**Figure 4:**
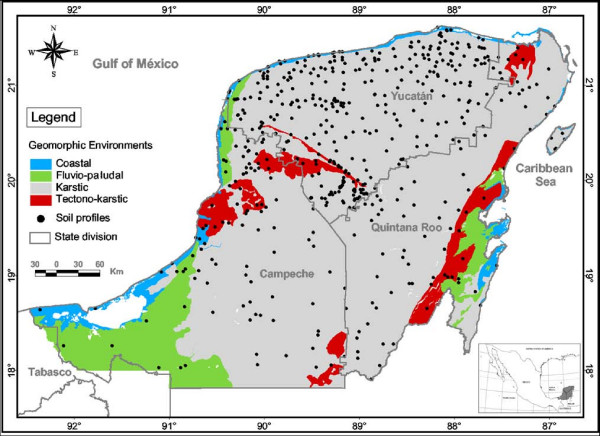
**Geomorphic environments in the Yucatán Peninsula (southeast México)**.

The meaning of some Maya soil names may vary throughout the Yucatán peninsula. Such is the case of the Ak'al che', for instance. These soils can be Gleysols as in Campeche or Stagnosols as in some places of the southern Yucatán state. The difference between gleyic and stagnic properties is taken care of in the Maya soil classification by adding primary and secondary qualifiers to the central concept of the soil group. In general, interregional variations such as in the above example are more common than intraregional variations. However, it can be assumed that the Maya soil classification applies to a large part of the peninsula of Yucatán (ca 152,000 km^2^) for two main reasons. One is the spatial repetition of four geomorphic systems all over the area: coastal, karstic, tectono-karstic, and fluvio-paludal, each one showing specific soil-relief patterns [12,14,25]. Our study documents the soils found in these four geomorphic environments and describes their variability over an area of nearly 39,000 km^2 ^(Figure 4). This can be considered a representative sample of the peninsula. The second reason is linguistic homogeneity as 1.5 million people speak the Yucatec Mayan language in the Yucatán peninsula [51,52]. Obviously, additional studies are needed to improve the MSC and test its applicability in a variety of settings throughout Yucatán.

Soil heterogeneity at parcel level is well recognized by Maya peasants who select the type of milpa according to soil quality and variability. For instance, in the center of the Yucatán state, several types of milpa are used including slash-and-burn milpa and sugar cane milpa, but intensive milpa is practiced only on K'an kab lu'um and Chak lu'um soils, using manure, manual tillage, and cover crops with herbaceous legumes. In Tzek'el, Ch'ich' lu'um and Ch'och'ol lu'um, the planting distance is 1 × 1 m, using a local maize variety along with beans and squash. Whereas in Chacklu'um and K'an kab lu'um, the planting distance is 0.6 × 0.6 m with an improved variety of maize together with sweet potato and cassava [29]. This local soil variability should be reflected in soil maps using the MSC as a reference system.

## Conclusions

The conclusions about the Yucatec Maya soil knowledge that can be derived from this study are as follows: (a) the identification of soils in the Yucatec Maya classification may be made using a key similar to that used in the WRB; (b) the MSC is a natural system based on key properties, such as rock types, size and quantity of stones, color of topsoil and subsoil, depth, relief position, water dynamics, and plant-supporting processes; (c) the MSC addresses the soil properties of surficial and subsurficial horizons that have morphological, genetic and practical importance; (d) the soil properties used in the MSC can help generate primary and secondary qualifiers for the WRB (e.g., Chaltunic, Ch'och'olic, Ch'ich'ilic). However, much effort is still needed to go deeper into the Maya soil knowledge. In particular, a better understanding of the diagnostic properties used and their relationships with soil forming factors is necessary, before a complete classification system can be established, especially at the lower categorical levels.

## Competing interests

The authors declare that they have no competing interests.

## Authors' contributions

FB carried out the soil surveys, peasant interviews and the building of the first version of the Maya soil classification. JAZ improved the Maya soil classification and reviewed previous versions of the paper. FB and JAZ wrote the final version of the paper.
